# Opportunities, Challenges, and Prospects in Electrochemical Biosensing of Circulating Tumor DNA and Its Specific Features

**DOI:** 10.3390/s19173762

**Published:** 2019-08-30

**Authors:** Susana Campuzano, Verónica Serafín, Maria Gamella, María Pedrero, Paloma Yáñez-Sedeño, José M. Pingarrón

**Affiliations:** Departamento de Química Analítica, Facultad de CC. Químicas, Universidad Complutense de Madrid, E-28040 Madrid, Spain

**Keywords:** electrochemical biosensor, circulating tumor DNA, cancer, mutations, epigenetic changes, liquid biopsy

## Abstract

Nowadays, analyzing circulating tumor DNA (ctDNA), a very small part of circulating free DNA (cfDNA) carried by blood, is considered to be an interesting alternative to conventional single-site tumor tissue biopsies, both to assess tumor burden and provide a more comprehensive snapshot of the time-related and spatial heterogeneity of cancer genetic/epigenetic scenery. The determination of ctDNA and/or mapping its characteristic features, including tumor-specific mutations, chromosomal aberrations, microsatellite alterations, and epigenetic changes, are minimally invasive, powerful and credible biomarkers for early diagnosis, follow-up, prediction of therapy response/resistance, relapse monitoring, and tracking the rise of new mutant subclones, leading to improved cancer outcomes This review provides an outline of advances published in the last five years in electrochemical biosensing of ctDNA and surrogate markers. It emphasizes those strategies that have been successfully applied to real clinical samples. It highlights the unique opportunities they offer to shift the focus of cancer patient management methods from actual decision making, based on clinic-pathological features, to biomarker-driven treatment strategies, based on genotypes and customized targeted therapies. Also highlighted are the unmet hurdles and future key points to guide these devices in the development of liquid biopsy cornerstone tools in routine clinical practice for the diagnosis, prognosis, and therapy response monitoring in cancer patients.

## 1. Introduction

Cancer is one of the leading causes of death worldwide. According to the World Health Organization (WHO) statistics, cancer accounts for 1/6 of the global death toll, and more than 40% of cancers can be overcome if they are diagnosed early [1].

During the last few years, the assessment methods for cancer patients are shifting from actual decision-making, based on patients’ clinic-pathological features to the biomarker-driven treatment strategies, relying on genotypes and customized targeted therapies. Being aware that intra-tumoral heterogeneity leads to temporal clonal evolution induced by therapy, which can lead to the development of acquired resistance to therapies that used to work effectively, active checking of tumors clonal genetic composition has been progressively recognized as a cornerstone procedure for improving patient results and their life quality. In this context, liquid biopsies can be used, not only for real-time monitoring of disease and therapies response, but also to identify the development of strong subclones [2]. Besides interrogating the active alterations in circulating tumor DNA (ctDNA) levels, which is the fraction of hematogenous circulating-free DNA (cfDNA), specifically originating from tumors, the analysis of altered biomarker patterning in ctDNA may serve as a preliminary quantitative metric for analyzing the full tumor genome and tracking drug response, and/or therapy resistance [2]. 

ctDNA analysis outperforms imaging techniques and the gold standard single-site conventional tumor tissue biopsies, which are not informative in early stages of development or recurrence stages, and in most cases may not represent the diversity of DNA changes [3,4]. Moreover, puncturing is used to obtain pathological sections but is not adequate for some patients who have undergone surgery and some traumas, and also can cause distress and accidental fall-away of tumor cells from the tumor tissues, leading to tumor metastasis. Furthermore, ctDNA may still be present and detected in hardly detectable tumors, such as metastatic or too small ones, or after their recent excision, having a half-life in vivo of just a few hours. Despite single-site solid biopsy, the feasibility for repeating ctDNA sampling in blood allows the spatial and temporal characteristics of the disease to be examined, thereby providing a wider snapshot of intra-tumor clonal diversity, compared to biopsied material. Furthermore, the ‘real-time’ monitoring of patients following surgery [3,5] and/or longitudinal studies of on-treatment patients as a readout of therapeutic efficacy [6] can be carried out. Moreover, since patients undergoing cancer therapy are routinely monitored by blood tests assaying circulating protein biomarkers, the incorporation of ctDNA analysis into patient care and management, with only very minor changes in clinical practice, would be of great interest [3]. 

Liquid biopsy is very suitable for the early detection of tumors because tumor biomarkers are freed and circulate in the blood in the initial steps of a tumor. The interrogation of tumor-specific mutations, and copy number variations, of ctDNA are among the preferred tumor biomarkers for real-time ‘tracking’ of cancer patients on account of the ctDNA presence in the blood of early tumor patients [2,3]. Recent studies have highlighted that ctDNA concentrations are substantially higher in cancer patients compared to healthy controls, although they may be affected by different variables (tumor stage, grade, aggressiveness, etc.). Therefore, ctDNA provides a more reliable diagnostic when used in combination with other cancer-associated surrogate markers, including tumor specific molecular aberrations (gene mutations and fusions, chromosomal rearrangements), microsatellite, methylation patterns alterations (hipermethylation and hypomethylation events both at locus-specific and global levels), cancer-derived viral sequences, and the amplification of specific genes [2,7]. The identification and determination of these reliable and personalized associated markers are greatly significant in diagnosing cancer in its early stages, monitoring tumor progression, predicting risk of relapsing, tracking the rise of new mutant subclones, and assessing drug response/resistance, thus guiding effective therapeutic interventions, and improving patient outcomes (overall and disease-free survivals) [2]. However, since ctDNA might make up as low as 0.01% of cfDNA, its determination, particularly at the early stages of tumor development, is a daunting task. 

Currently, ctDNA analysis is made mostly using sequencing-based technologies and PCR-based methods, as well as other advanced detection systems, including mass-spectrometry (MS), matrix-assisted laser desorption/ionization (MALDI), and surface-enhanced Raman scattering (SERS) [1,2,5,8,9]. However, these techniques are time-consuming and costly for their real accomplishment in a diagnostic set [2]. Electrochemical biosensors show remarkable features for the detection of ctDNA compared to these mentioned techniques, because of their great sensitivity, specificity, portability, plain use, rapid readout, and amenability to high levels of multiplexing and direct analysis in complex biological matrices. Moreover, they are simple to fabricate, straightforward to automate and implement using cost-effective instrumentation, which make them applicable in point-of-care (POC) systems and/or undeveloped areas [1,10,11]. 

**Scheme 1:**
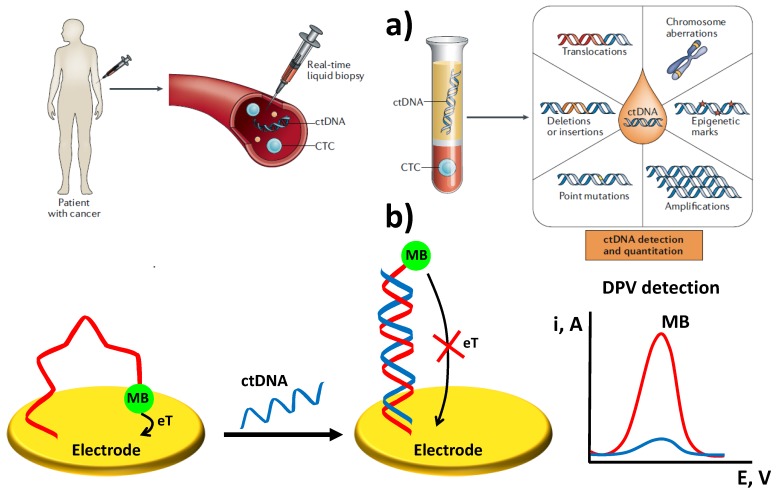
Schematic illustration of liquid biopsy, ctDNA and specific features (**a**) and a type of electrochemical DNA sensor for ctDNA determination (**b**). Figure reprinted and adapted from [12] with permission (**a**) and drawn based on [13] (**b**).

Considering the rapidity with which this field moves, after a brief introduction of the main features of ctDNA as minimally invasive cancer biomarker, as well as its most common detection strategies, this review article discusses recent trends and opportunities offered by electrochemical biosensing in the analysis of ctDNA and its specific features (Scheme 1). The main hurdles to overcome and future directions in this field are also discussed. 

## 2. Circulating Tumor DNA: Properties, Specific Features and Clinical Applications

Cancer is essentially a genetic disease, and ctDNA englobing combined genetic material from the primary tumor and metastases, is considered key to tumors in blood and closely connected with their occurrence, heterogeneity, and growth. ctDNA clinical values are used for: early detection, monitoring of tumor dynamics, evaluation of treatment efficacy, streamlining drug development, prediction of cancer recurrence, assessment of molecular heterogeneity, and monitoring of minimal residual disease after curative therapies [1,3,5,6,14].

Above other circulating markers, such as tumor cells or cancer-related protein biomarkers, ctDNA offers a range of analytical advantages including, (i) is liberated from tumor cells in many sick areas to the circulatory system thus minimizing sampling bias [9]; (ii) incorporates tumor-specific genetic alterations (single nucleotide mutation, microsatellite, copy number, methylation modifications, etc.) that ensure its specificity as a tumor biomarker; (iii) is more highly fragmented (100–180 bp) than wildtype cfDNA [5,7]; (iv) occurs mainly as double-stranded DNA (ds-ctDNA) [1]; (v) occurs in peripheral blood that may be excreted in urine, sputum or feces.; (vi) its level in cancer patients is more elevated than in healthy people, although it depends on many tumor variables (location, size, and vascularity), types, and stages of the disease [5]; (vii) its ratio to cfDNA in peripheral blood vary from as little as 0.01% up to 90% [3] (cfDNA ranges from 0 to 100 ng mL^−1^ in healthy individuals and can be up to 1000 ng mL^−1^ in cancer patients [8]); (viii) its half-life in blood is short, from about ten minutes to 2–2.5 h [1,6,8] (its clearance is believed to happen through the kidneys or liver, or by nucleases degradation [5]). Indeed, this unfavorable stability of ctDNA, although adversely affecting the sensitivity of the detection, is advantageous for real-time monitoring of the tumor status [3,9]. 

Going ahead in the high clinical relevance of ctDNA, its levels have been shown to be associated with poor outcomes, such as a reduced overall survival (OS) rate and disease-free survival (DFS) rate in diverse cancers. However, most of the research studying the potential of ctDNA analysis is devoted to the detection of point mutation/s in one or a set of cancer specific genes. In contrast to the somatic point mutations, which may be shared by diverse tumor types, chromosomal rearrangements are greatly tumor-specific and thus may represent a distinct “fingerprint” to be exploited for cancer detection, as well as in predicting the development of metastasis and post-surgery reappearance [2]. Relative variations in the fractional concentration of diverse tumor mutations in ctDNA may suggest a clonal evolution and, therefore, be associated with the rising resistance to therapy, months before cancer progression is clinically evident. Moreover, since plasma ctDNA levels represent total systemic tumor load, variations in ctDNA levels post-surgery are related to the extent of surgical resection. These ctDNA levels are reduced after the removal of the entire tumor and may rise again as soon as new lesions appear. Therefore, the analysis of ctDNA genomic profiles may be very helpful in revealing tumor dormancy. All these findings emphasise the value of minimally invasive ctDNA analysis for dynamic genetic checking of cancers, and the importance of identifying and foreseeing the advent of therapy resistance [2].

However, the high fragmentation and extremely low ctDNA concentration in a large background of wildtype circulating DNA, and the fact that clinically significant target sequences frequently diverge from the wildtype types in a single DNA base pair (bp), make ctDNA determination, and its screening for characteristic features, particularly challenging [5,8,9]. 

## 3. Conventional Methodologies for the Determination of ctDNA and Characteristic Signatures

Current technologies for ctDNAs analysis can be classified in targeted (monitoring single or few tumor-specific mutations or at locus-specific regions) and untargeted (genome-wide analysis for copy number aberrations, point mutations and methylation events) methods. Although targeted monitoring requires detailed information about the tumor genome, it is usually extremely sensitive, as mutations can be detected at an allele frequency of 0.01% with high specificity. The advantages of untargeted strategies, with an overall low sensitivity (5–10%), involve no requirements for previous information about the primary tumor’s genome or the ability to identify changes (novel candidate biomarkers) occurring during tumor treatment.

Common methods for analyzing ctDNA have been recently reviewed by Rodda et al. [5]. They include qualitative sequencing-based technologies (deep sequencing, next-generation sequencing, NGS, tagged-amplicon sequencing, TAm-Seq, massively parallel sequencing, MPS), and quantitative PCR-based methods (BEAMing, COLD-PCR, droplet digital-PCR and real-time PCR), as well as other advanced techniques, such as MS, MALDI, and SERS [1,2,5,8,9]. 

These methods have allowed significant progress in ctDNA detection, using low amounts of input material and providing high sensitivity and specificity. However, some key drawbacks limit their application and partly restrict the exploitation of ctDNA in clinical environments: they involve complicated operations and slow turnaround times (sequencing-based approaches), being also liable to sequence-specific amplification biases, and need sample pre-treatment, thus remarkably raising the cost and time of analysis (PCR-based methods), and may be prohibitively expensive for optimal translational usage (sequencing-based and MS methods). Consequently, low-cost and portable platforms, entailing straightforward processes and capable of accurately detecting specific ctDNA mutations in untreated serum or blood, are best adapted for routine use in the clinic. In later years, the quick growth of electrochemical biosensors has born a novel advancement in ctDNA detection, making electrochemical biosensing platforms ideal candidates, due to their capacity for automation, amenability to high levels of multiplexing and sensitivity, affordability, user-friendliness, and minuscule amount of sample requirement [1,2]. Although, they have not yet been exploited in connection with electrochemical biosensors for ctDNA analysis, it is worth highlighting here the use of new functional nanomaterials, including peptide and protein amyloid-based nanostructures or graphene/biomolecules nanohybrids for biomedical applications [15,16,17,18,19].

Table 1 outlines significant characteristics of electrochemical biosensing approaches described in the last 5 years for ctDNAs determination. These methods are discussed in more detail in the following sections, through a classification, based on the target feature of ctDNA detected.

## 4. Electrochemical Biosensing of ctDNA and Surrogate Markers

### 4.1. Methods for ctDNA Quantification

**Figure 1:**
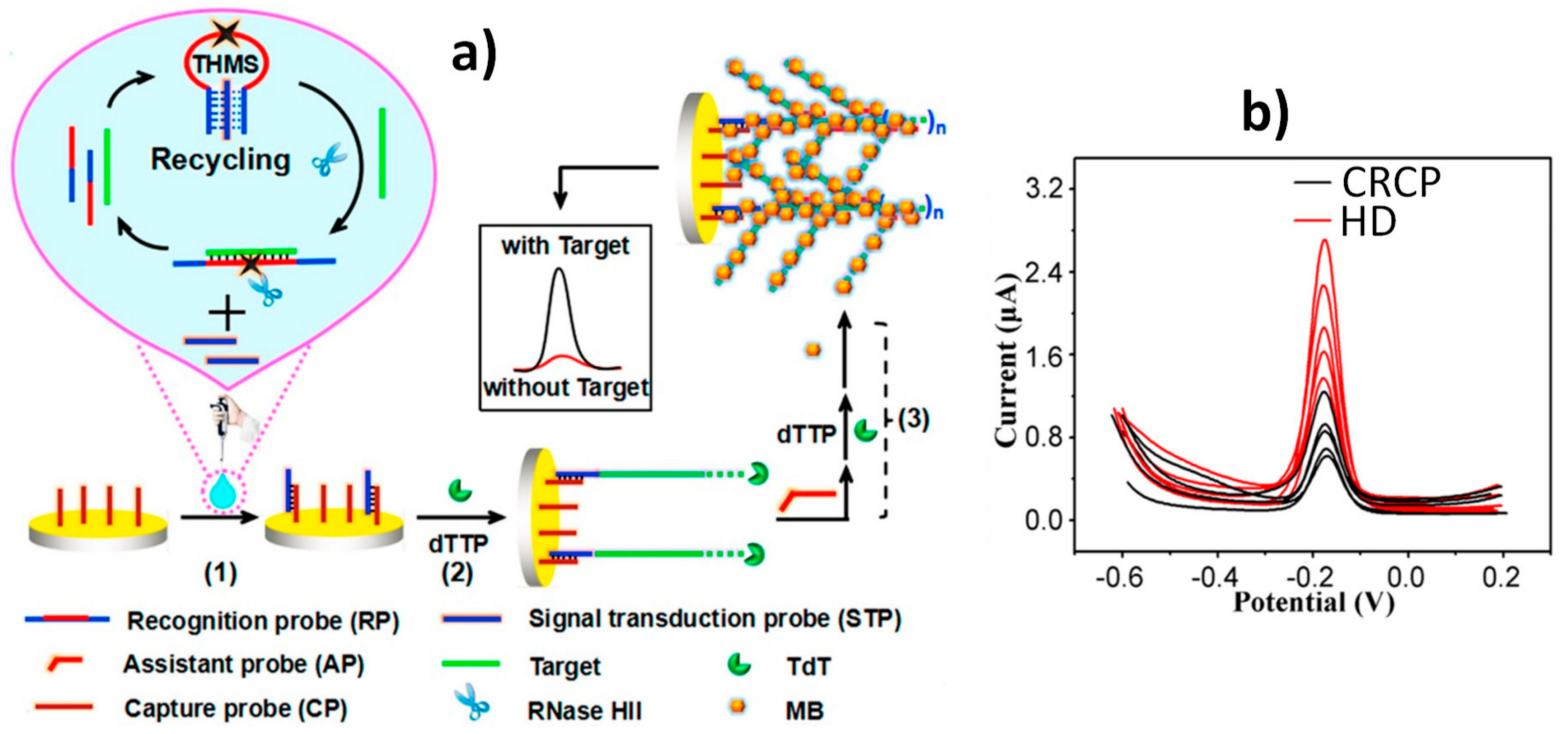
(**a**) Schematic illustration of the biosensor developed for the detection of KRAS G12DM ss-ctDNA involving the use of a THMS probe and TdT and Rnase HII dual amplification. (**b**) DPV traces recorded with the developed biosensor in the analysis of DNA extracted from plasma samples of 5 HDs and 5 CRCPs. Reprinted and adapted from [20] with permission.

Wang et al. [20] proposed a versatile electrochemical biosensor, using a dual enzyme assisted multiple amplification strategy for the ultrasensitive detection of KRAS G12DM ss-ctDNA. The biosensor involved the use of molecular recognition (a triple helix molecular switch, THMS), and signal transduction probes. Furthermore, the terminal deoxynucleotidyl transferase (TdT) and ribonuclease HII (RNase HII) dual enzyme assisted multiple amplification to form a dendritic structure (see Figure 1a). The presence of target ctDNA opened the THMS and triggered Rnase HII-assisted homogenous target recycling amplification to produce a substantial signal transduction probe (STP). The released STP, hybridized with the capture probe, was immobilized on a gold electrode and triggered the first-step TdT-mediated extension to form the DNA “trunk”. Then, the specially designed assistant probe (AP), which partially hybridized with the DNA “trunk”, was further introduced to initiate the second-step TdT-mediated extension and generated a long stable DNA dendritic structure, which would be decorated with a high number of methylene blue (MB) molecules, used as signal reporters, producing a significant amplification in the electrochemical response. Using as analytical readout the MB DPV oxidation peak, and combining the efficient recognition of the designed THMS with the excellent multiple amplification ability of Rnase HII and TdT, this sensing platform was able to determine synthetic KRAS G12DM target DNA over the wide range 0.01 fM-1 pM, with a limit of detection (LOD) of 2.4 aM. The biosensor was applied to determine ctDNA in DNA extracted from plasma of colorectal cancer patients (CRCP) and healthy donors (HD). DPV traces, shown in Figure 1b, confirmed the higher expression of the KRAS G12DM in the CRCP plasma with mean concentrations of 0.19 fM and 11.59 aM in plasma from CRCP and HD, respectively. Importantly, this biosensing strategy can be readily extended to detect a wide range of ctDNA simply by substituting the THMS loop for different sequences.

### 4.2. Electrochemical Biosensing of Tumor-Specific Mutations

**Figure 2:**
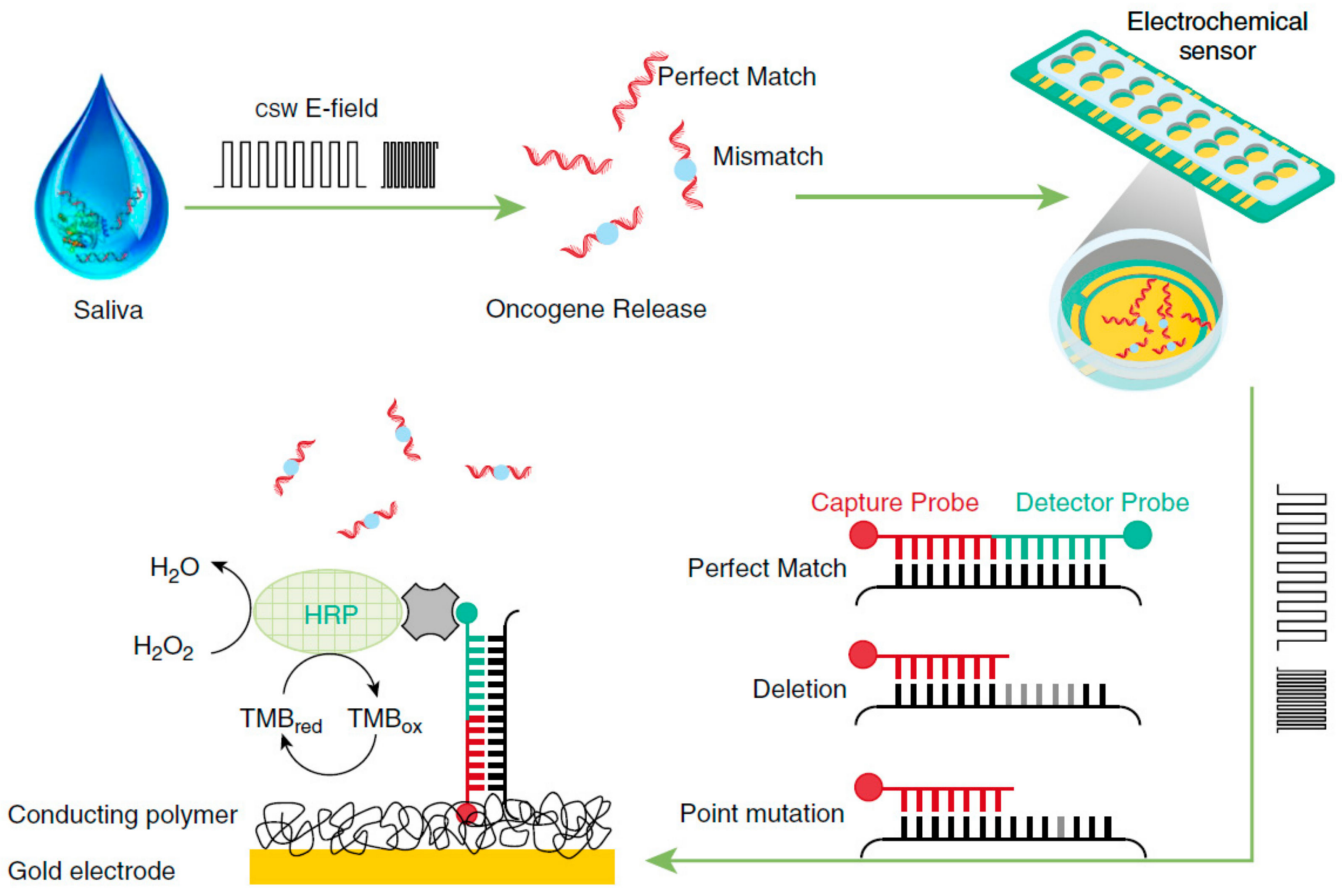
Multiplexed electrochemical sensor in connection with EFIRM technology for the determination of *EGFR* mutations in bodily fluids of patients with lung cancer. Reprinted from [21] with permission.

Wei et al. [21] proposed the use of electric field–induced release and measurement (EFIRM) technology, in connection with a multiplexed electrochemical biosensor, for the determination of epidermal growth factor receptor (*EGFR*) mutations in the saliva and plasma of non-small cell lung carcinoma (NSCLC) patients. The method used cyclic-square waves of the electrical field (csw E-field) to release the genetic material from the biofluid. The determination involved a sandwich hybridization format with paired capture and detector probes specific for the two target mutations detected (exon 19 deletion and L858R point mutation). The capture probe-modified electrochemical sensor consisted of a conducting polymer-based electrochemical chip with an array of 16 bare gold electrode chips). After labeling the fluorescein isothiocyanate (FITC)-modified detector probe with Fab fragments from an anti-fluorescein antibody conjugated to horseradish peroxidase (HRP-anti-FITC Fab fragments), the chronoamperometric signals were measured in the presence 3,3´,5,5´-tetramethylbenzidine (TMB) and H_2_O_2_ at -200 mV versus the Au pseudo-reference electrode (Figure 2). It is remarkable that this method required less than 10 min, and just 20-40 µL of biological sample, to perform the determination.

Electrochemical DNA sensors, used for the detection of specific mutations in the p53 tumor suppressor gene (*TP53*), were reported by Esteban-Fernández de Ávila et al. [22], through the covalent immobilization, via carbodiimide chemistry, of an amino and biotin dually-labeled hairpin-specific DNA capture probe onto SPCEs, which were functionalized with a water-soluble reduced graphene oxide−carboxymethylcellulose hybrid nanomaterial. The immobilized hairpin probe, initially in the folding state, was selectively opened in the presence of the target DNA (specific region of the non-mutated *TP53*). After enzymatic labeling of the hairpin probe with a streptavidin-horseradish peroxidase conjugate, amperometric responses were recorded in the presence of TMB/H_2_O_2_. The signals were inversely proportional to the concentration of the target DNA (wild-type *TP53*) because of the remarkably increased distance between the attached enzymatic conjugate and the electrode surface. The bioplatform provided a LOD of 2.9 fmol for the synthetic target DNA and allowed a clear discrimination towards the single base mutated *TP53* sequence (missense mutation G → A in codon 175 leading to cancer-triggering deactivation of the tumor suppressor protein p53). The biosensor was applied to spiked untreated human serum and saliva, and was able to analyze the endogenous *TP53* status in only 50 ng of cDNA, synthetized by reverse transcriptase from total RNA (RNA_t_), which was extracted from one non-tumorigenic epithelial (MCF-10A) and two cancer (MCF-7 and SK-BR-3) human breast cells.

Label-free and low-fouling biosensors for breast cancer susceptibility gene (*BRCA1*), was reported by Wang et al. [23], by modifying a glassy carbon electrode with highly cross-linked polyethylene glycol film containing amine groups and self-assembling of AuNPs and immobilization of a thiolated DNA capture probe. The increase in the electron transfer resistance measured by electrochemical impedance spectroscopy (EIS) in the presence of [Fe(CN)_6_]^3-/4-^ after hybridization with the target DNA resulted in a linear range from 50.0 fM to 1.0 nM, with a LOD of 1.72 fM and a clear discrimination against a single-base mismatched sequence. The label-free DNA sensor was used to analyze *BRCA1* in spiked serum samples.

**Figure 3:**
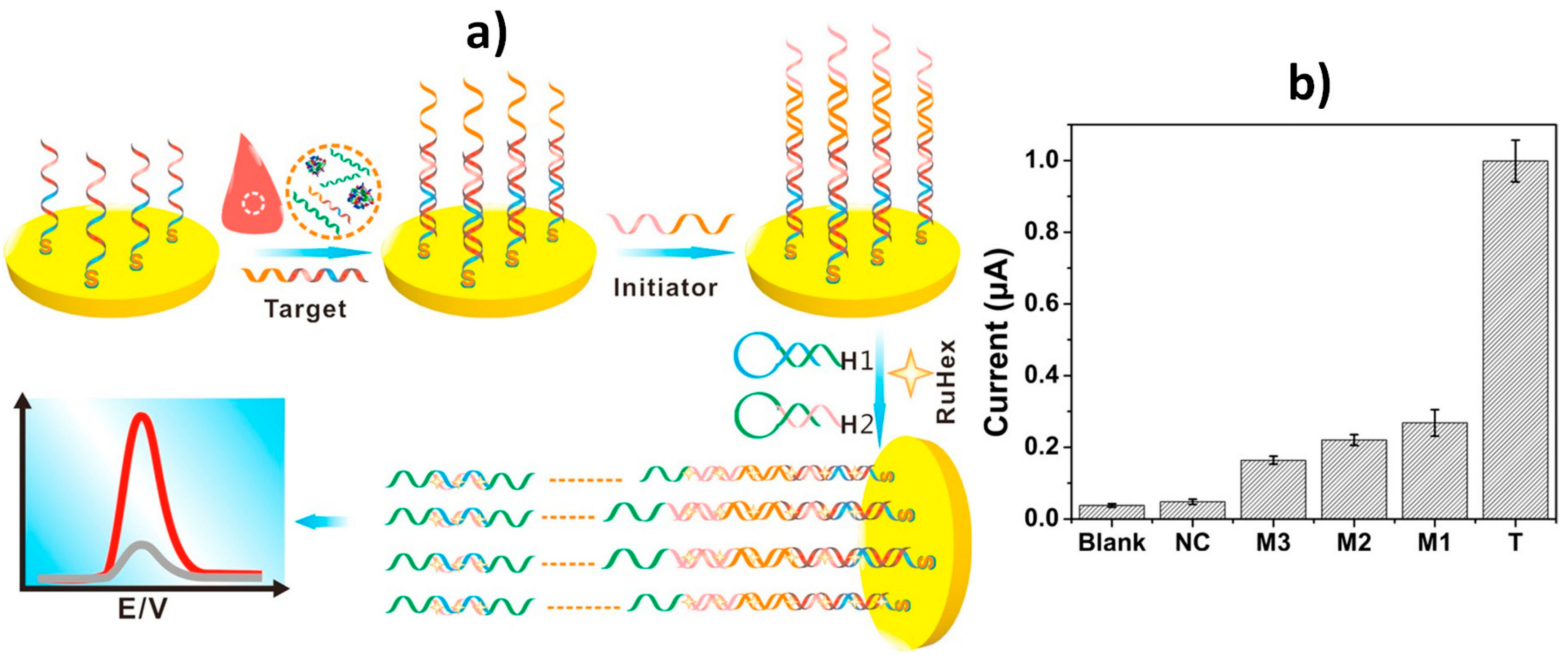
(**a**) Schematic illustration of the HCR-based DNA sensor for *BRCA1* determination. (**b**) DPV responses provided by the DNA sensors towards 20 pM of synthetic sequences of the target DNA (T), single-base (M1), two-base (M2), three-base (M3) mismatched and non-complementary (NC) sequences. Reprinted and adapted from [24] with permission.

Yang et al. [24] reported another DNA electrochemical sensor for *BRCA1* using hybridization chain reaction (HCR) (Figure 3a). HCR is as an isothermal amplification methodology, which does not require complicated variable temperature programs nor the participation of enzymes. It uses two supplementary nucleic acid probes (H1 and H2), which can steadily coexist in solution with no initiator probe, but their loops are alternately opened to form dsDNA when the initiator is present [33]. The DPV signal of RuHex, used as electrochemical redox indicator provided a linear correlation with the logarithm of the target sequence concentration between 1 aM and 10 pM with a LOD of 1 aM. In addition, this HCR-based DNA sensor was able to discriminate a single mismatched sequence (M1 in Figure 3b) and to detect low concentrations of the target synthetic DNA (10 pM) in spiked 50% human serum.

**Figure 4:**
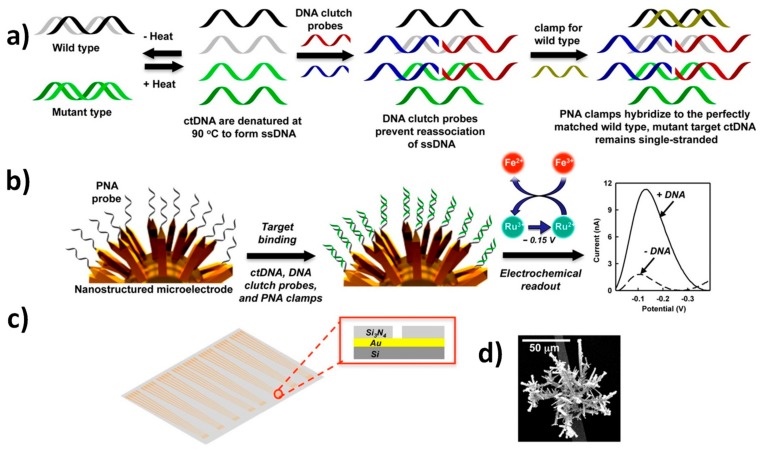
(**a**) Schematic diagram of the clutch probe strategy for ctDNA detection, (**b**) hybridization of the sample onto PNA-probes-modified NMEs and DPV electrochemical detection, (**c**) sensor chip layout and cross-section of the chip at aperture (inset) and (**d**) scanning electron microscopy image of the nanostructured electrode. Reprinted and adapted from [25] with permission.

An attractive electrochemical assay, able to directly detect mutation within *KRAS* and *BRAF* in ctDNAs, from serum of cancer patients with no need for enzymatic amplification, has been proposed by O. Kelley group [25]. This approach employed a peptide nucleic acid (PNA) wild-type blocker, together with DNA “clutch probes” (DCPs), which are pairs of ssDNA molecules that hinder the re-association of denatured ssDNAs. In this way, one of the two strands in dsDNA molecules was available for hybridization, and deactivated strongly correlated sequences available in solution, thus promoting preferential association of mutated sequence-based hybridization events, with finely nanostructured microelectrode (NME), functionalized with allele-specific PNA probes (Figure 4). The NME was prepared using a SiO_2_-coated silicon wafer to construct contact pads and electrical leads. Then, a Si_3_N_4_ layer was placed, in order to passivate the surface and 5 μm apertures. These were then opened in the top passivation layer by photolithography to supply a template for the growing of electrodeposited Au. The as-formed Au structures were then coated with a thin layer of Pd. The three-dimensional nanostructure of the NME allowed the probes to have an adequate alignment direction, thus increasing the hybridization efficiency. After target hybridization, the biosensors were interrogated, using an electrocatalytic reporter pair of Ru(NH_3_)_6_^3+^ and Fe(CN)_6_^3−^, by DPV. The voltammetric response increased in the presence of the target mutant. Ru(NH_3_)_6_^3+^ was electrostatically attracted to the negatively-charged phosphate backbone of nucleic acids that binds to the probes immobilized on the surface of electrodes, and was reduced to Ru(NH_3_)_6_^2+^ when the electrode was biased at the reduction potential [34]. The Fe(CN)_6_^3−^, present in solution, chemically oxidized Ru(NH_3_)_6_^2+^ back to Ru(NH_3_)_6_^3+^, which allowed for multiple turnovers of Ru(NH_3_)_6_^3+^ and generated a high electrocatalytic current (Figure 4c). The method was able to distinguish 1 fg μL^−1^ of a selected mutation within 100 pg μL^−1^ of wild-type DNA, i.e., it detected 0.01% mutations and allowed for the accurate analysis of ctDNAs, extracted from serum samples, that were collected from lung cancer and melanoma patients.

### 4.3. Electrochemical Biosensing of Methylation Changes in ctDNA

Cancer-specific ctDNA methylation can be used to quantitate tumor DNA, providing information about the tumor burden level. Moreover, tumor-specific methylation is less variable across tumors than mutations, so that methylated ctDNA can be a marker of surgery outcome, treatment response, tumor aggressiveness, likelihood of recurrence, and patient survival [3]. 

The modification of cytosine © to 5-methylcytosine (5-mC) by DNA methyltransferases is the most studied epigenetic alteration, due to its important role in carcinogenesis. Apart from 5-mC, the Ten-Eleven Translocation (TET) enzymes mediate sequential oxidation of 5-mC to 5-hydroxymethylcytosine (5-hmC), then to 5-formylcytosine (5-fC), and finally to 5-carboxylcytosine (5-caC) [35]. These intermediates in the demethylation pathway have been shown to confer unique transcriptional function to genes and are considered important indicators of the initiation and progression of cancers. Locus-specific DNA hypermethylation of relevant tumor suppressor genes at the promoter region provokes their silencing and, accordingly, this mechanism has been proposed as an early diagnostic biomarker that is strongly associated with tumor aggression. Moreover, it can be leveraged to monitor the success of the administered therapies, alterations of the cytosine epimarks at the global level, and used for the identification of cancer patient subsets, that might engender distinct clinical outcomes. In addition, the reduction in the circulating levels of 5-hmC, 5-fC, and 5-caC, is tightly associated with solid tumor development [36]. Therefore, effective and flexible strategies to differentiate between these cytosine epimarks, both at user-defined sequence positions and global levels, are highly sought. Electrochemical biosensing strategies, involving both, amplification-free or amplification strategies, are gaining attraction for the determination of these biomarkers. They are also envisioned as promising alternatives to overcome some of the most important drawbacks posed by current conventional methodologies. 

The electrochemical analysis of circulating methylated DNA in plasma was proposed by Chen et al. [26], implementing a paired-end tagging amplifications strategy. Two DNA primers, each labeled with digoxigenin (Dig) and biotin, were designed for the recognition and amplification of methylated DNA. Paired-end tagging amplicons and avidin-HRP molecules were successively captured on the electrode surface, modified with anti-Dig antibody, and chronoamperometric detection was carried out in the presence of TMB/H_2_O_2_. The method allowed the detection of as little as 40 pg of methylated genomic DNA, 1% methylation level, and tumor-specific methylated DNA in plasma of NSCLC patients, attaining a very high clinical sensitivity of 91%. 

**Figure 5:**
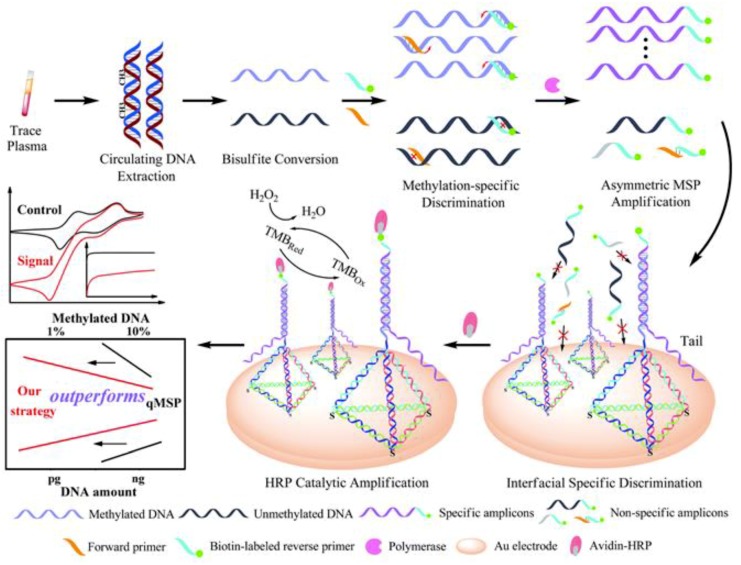
Chronoamperometric determination of circulating methylated DNA at single copy level involving bisulfite treatment, aMSP and efficient hybridization of the specific ss-amplicons on a gold electrode modified with tetrahedral DNA nanostructures. Reprinted from [28] with permission.

Wang et al. [28] proposed a similar approach in which the sequential discrimination–amplification scheme was employed for the detection of circulating methylated DNA with single-copy sensitivity. The methodology integrated bisulfite treatment, asymmetric methylation-specific PCR (aMSP), and efficient hybridization of the resulting ss-amplicons on a gold electrode, modified with tetrahedral DNA nanostructures (Figure 5). The use of tetrahedral DNA probes greatly improved the assay performance, in contrast with the use of conventional thiolated probes, since they notably increased target hybridization and reduced the non-specific adsorption of amplification by-products, due to the fixed scaffold, ordered orientation, and fully-regulated spacing. Using chronoamperometry with the HRP/H_2_O_2_/TMB system, the method allowed the successful examination of just a single methylated DNA molecule in the presence of a 1,000-fold excess of unmethylated alleles and was applied to plasma samples from lung cancer patients.

Daneshpour et al. [27] reported an electrochemical immunosensor for the analysis of *RASSF1A* promoter methylation, using Fe_3_O_4_/N-trimethyl chitosan/gold nanocomposite, as a tracing tag to label a DNA detector probe, and a SPCE modified with anti-5-mC antibody entrapped in thin polythiophene film for the selective capture of methylated DNA. The electrochemical detection implies oxidation of the Au nanoparticles and monitoring their subsequent reduction by DPV. The method exhibited a linear range from 1 × 10^−14^ M to 5 × 10^−9^ M and a LOD of 2 × 10^−15^ M for the synthetic methylated target DNA. Moreover, successful recoveries were reported in the analysis of plasma samples, spiked with increasing concentrations of synthetic methylated target DNA.

**Figure 6:**
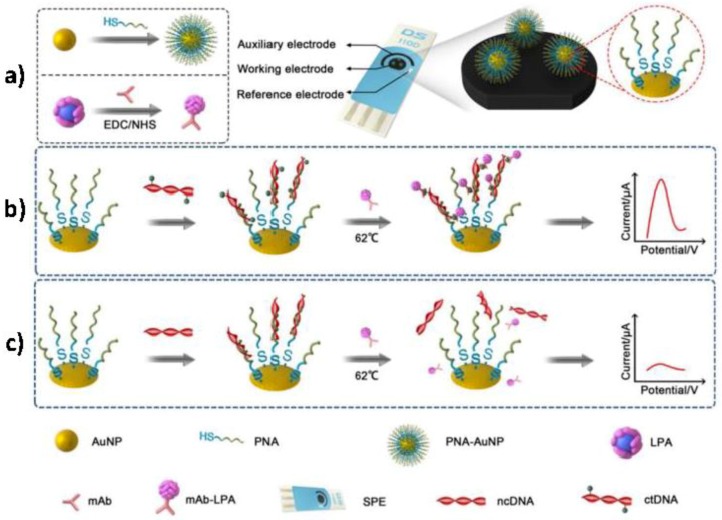
Schematic displays showing the preparation of PNA-AuNPs conjugates and LPA-anti-5-mC bioconjugates (**a**) and the ctDNA (**b**) and ncDNA (**c**) detection at the constructed biosensor. Reprinted and adapted from [31] with permission.

Cai et al. [31] reported a PNA-AuNPs and lead phosphate apoferritin (LPA) nanoparticles-based dual biomarker detection platform to detect tumor-specific mutations and 5-mC methylation of *PIK3CA* gene in ctDNA (Figure 6a). A PNA probe and an anti-5-mC antibody were used to recognize the different parts of ctDNA, forming a sandwich-structure on a screen-printed electrode surface. AuNPs as nanocarriers to co-immobilize large loading of PNA probes and LPA were introduced for double signal amplification. As shown in Figure 6b, in the presence of ctDNA, a PNA-AuNPs/ctDNA/LPA-anti-5-mC sandwich was formed on a disposable SPE surface. Highly specific and sensitive detection of ctDNA was achieved by square wave voltammetry (SWV) from the lead ions released from apoferritin at an in situ plated bismuth film electrode. Conversely, ncDNA neither, matched perfectly with the PNA-AuNPs probe, nor reacted with LPA-anti-5-mC and, therefore, no sandwich structure was formed (Figure 6c). This DNA biosensor yielded a linear current response to ctDNA concentrations from 50 to 10,000 fM with a LOD of 10 fM and, it was successfully applied to detect ctDNA collected from serum of cancer patients.

**Figure 7:**
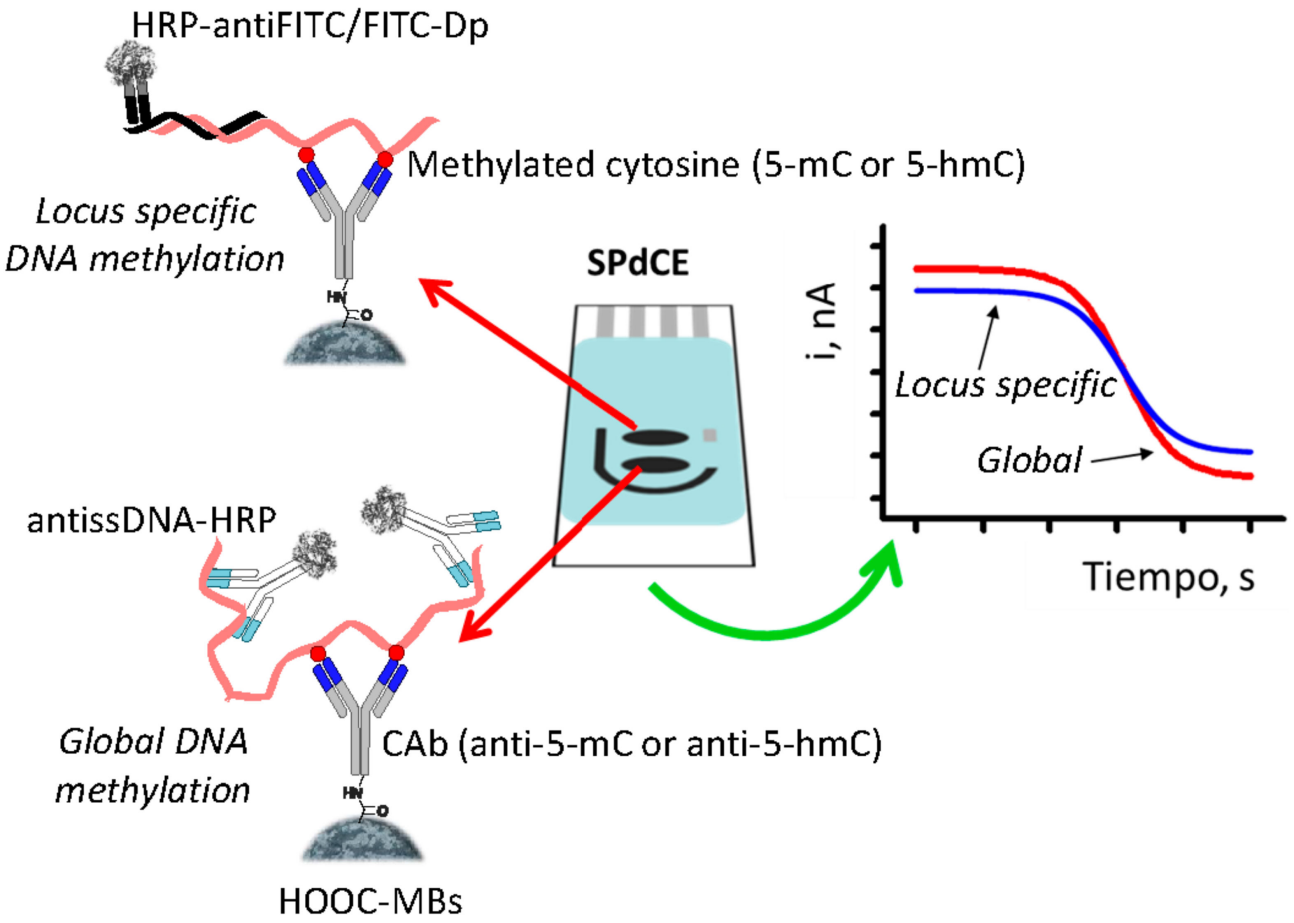
Schematic display of the dual electrochemical bioplatforms developed for the detection of 5-mC or 5-hmC both at global and gene-specific levels. Figure drawing based on [30].

Other simple and rapid strategies, not involving nanomaterials or nucleic acid-based amplification strategies, have been devised for the sensitive and selective detection of methylated ctDNA, such as those developed by Pingarrón´s group for the detection of 5-mC or 5-hmC, both at global and locus-specific levels. The strategies leant on the combination of functionalized magnetic microbeads (MBs), antibodies specific for the target methylated cytosines, and were used either to capture or detect the methylated DNA, specific biotinylated DNA probes, and amperometric detection at screen-printed carbon electrodes [29,30]. The developed methodologies achieved LODs of 0.43 and 1.2 pM for the synthetic 5-hmC, and 5-mC sequences of the *MGMT* tumor suppressor gene promoter region, respectively, in 45–90 min with a great reproducibility [30]. These bioplatforms were applied for the first time to the dual detection of the same or different kinds of epimarks both, at a global level and in different regions/loci of the same gene, or in different genes by using screen-printed dual carbon electrodes. Figure 7 shows that the assays included the effective capturing of the methylated ssDNA to MBs, modified with antibodies specific to the target epimark. The captured methylated DNA was labeled with a HRP-conjugated antibody, capable in detecting any ss-DNA sequence for global methylation, or with a fluorescein-5-isothiocyanate (FITC) labeled detector DNA probe, specific to the target methylated gene, and followed by conjugation with HRP-anti-FITC Fab fragments for locus-specific methylation. The developed bio-platforms were used for the determination of 5-mC methylations in the promoter region of *RASSF1A* gene, directly in serum samples from breast and lung cancer patients [30].

### 4.4. Electrochemical Biosensing of Cancer-Related Viral DNA Sequences

Common infections with particular viruses, such as human papillomaviruses (HPVs), are often cleared by the immune system without serious consequences. However, a small fraction of these infections become persistent over longer periods of time and are associated with an increased risk of developing pre-cancerous lesions, and ultimately cancer. On the other hand, there is evidence that people infected with Hepatitis B virus (HBV) are at high risks of developing liver cancer [32]. Accordingly, increased efforts have been made in recent years to develop and implement tests for cancer-related viral DNA sequences as screening options for early cancer diagnosis [37]. 

Ahangar and Mehrgardi [32] prepared an electrochemical DNA biosensor using a nano-porous gold electrode for the amplified detection of HBV. The method involved a sandwich hybridization configuration, with a thiolated capture probe immobilized on the nanoporous gold platform and an amino-labeled detector probe, which was further conjugated with ferrocene (Fc). The hybridization event was followed by monitoring Fc oxidation by DPV. The biosensor exhibited a dynamic range from 3 × 10^−5^ to 1 × 10^−3^ M, a LOD of 0.8 µM of target DNA, and was successfully employed for the determination of amplicons obtained from patients´ blood samples using limited PCR cycles.

**Figure 8:**
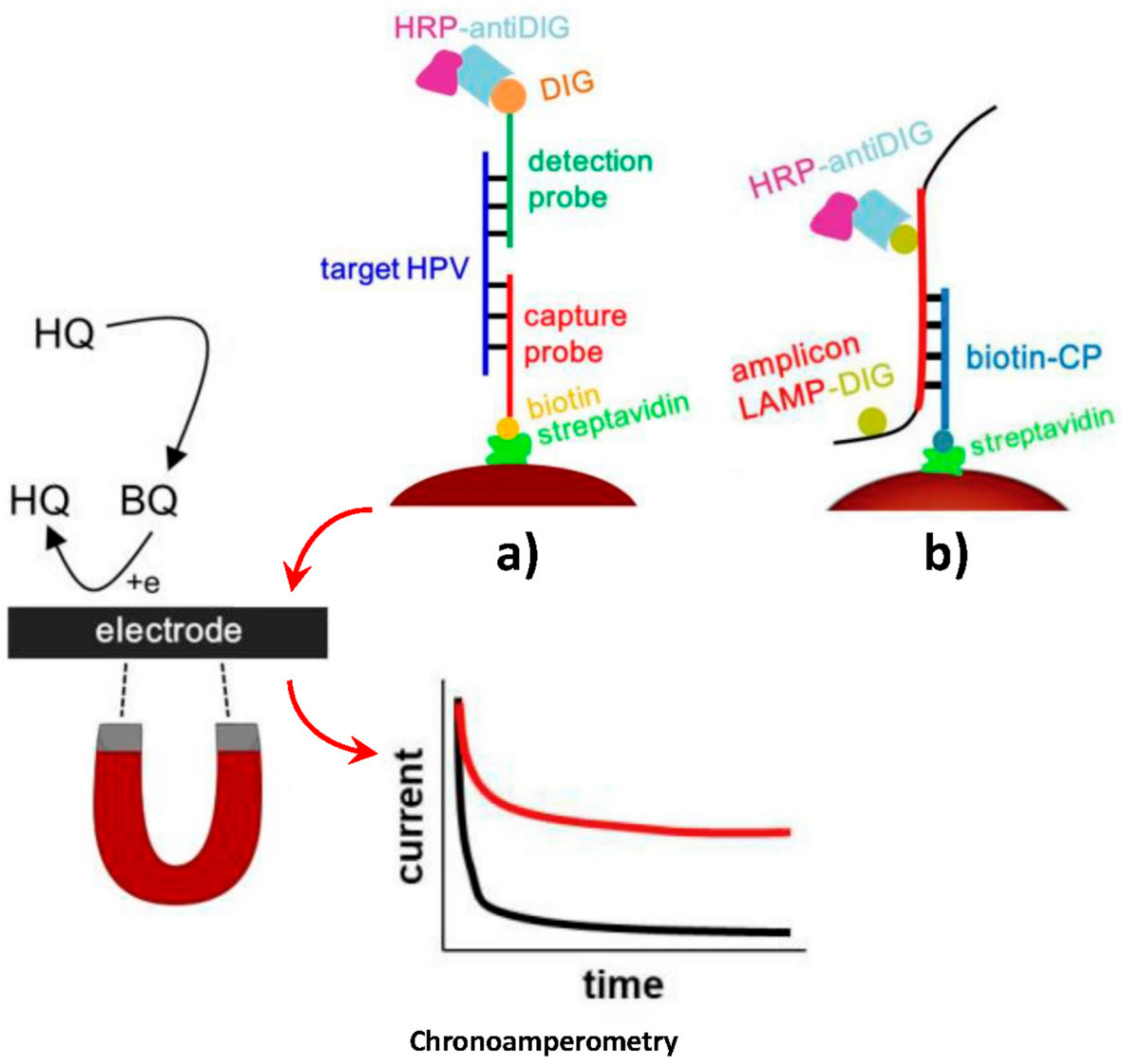
Schematic illustration of the MBs-based biosensing methods developed for the chronoamperometric determination of the synthetic HPV target DNA (**a**), and the DIG-labeled LAMP amplicon (**b**). Reprinted and adapted from (**a**) [38] and (**b**) [37] with permission.

Bartosik et al. [38] developed attractive MBs-based biosensing strategies for the detection of DNA sequences of HPV-16 and HPV-18, two of the most carcinogenic high-risk human papillomavirus (hrHPVs), which are responsible for about 70% of all cervical tumors. The approaches were implemented in connection with PCR [28] and loop-mediated isothermal amplification (LAMP) [38]. Both strategies involved the use of Strep-MBs, modified with a biotinylated DNA capture probe, to hybridize the synthetic target HPV DNA (Figure 8a) or the resulting amplicon (PCR or digoxigenin (DIG)-labeled LAMP one (Figure 8b). Once captured, the target DNA or the corresponding amplicon was sandwiched with a DIG-labeled detector probe. In both methods, enzymatic labeling was made with HRP-antiDIG antibody. Upon magnetic capturing of the modified MBs, the electrochemical transduction was performed by chronoamperometry, using the H_2_O_2_/HQ system, at a screen-printed electrochemical array comprising eight carbon electrodes. These methods yielded linear ranges from 1 pM to 1 nM, with a LOD of 1 pM, and from 0.1 to 50.0 ng and LODs of 0.1 and 0.7 ng, for the synthetic target DNA, and the DNA extracted from CaSki cells for the PCR, and LAMP-based methods, respectively. These MBs-involving biosensing strategies allowed the discrimination between these two hrHPVs and were used for the analysis of both, cancer cell lines and real patients’ cervical brush smears. 

## 5. General Conclusions, Insights, and Future Perspectives

Compared with other varieties of cancer-related biomarkers, ctDNA has an exclusive clinical value. Apart from the intrinsic advantages of employing liquid biopsies, such as handy sampling, low invasiveness, and real-time monitoring, its specificity is higher and can solve the problem of tumor heterogeneity [1]. Although, the current detection techniques, such as PCR and DNA sequencing, allow the detection of ctDNA, these techniques imply long detection processes, and are complex and expensive. Consequently, their real-world application is restricted. Simpler, faster, sensitive and specific electrochemical biosensors are emerging as powerful tools to detect ctDNA, also because of their commercial potential, since they are easy to make into portable and low-cost devices, or integrate in microfluidic chips usable by doctors and patients [1].

Apart from these unique features, the smart selection of bioreceptors, bioassay format, and amplification strategies, involving mainly the use of nanomaterials, nucleic acid amplification strategies, and multi-tags reagents, are key aspects to placing electrochemical biosensors in a privileged position for the analysis of ctDNA and their specific features. 

The methods discussed above show the involvement of unconventional probes such as THMS [20], DCPs [25], PNA [25,31] and tetrahedral DNA [28], and also the use of a wide variety of nucleic acid amplification strategies, including aMSP [28], TdT, and RNase HII amplification [20], HCR [24], PCR [37], and LAMP [38]. Moreover, to meet the challenging characteristics, required in terms of sensitivity and selectivity, some of the developed methods involve the smart coupling of different amplification strategies within the same bioplatform [20]. Other amplification strategies, explored for ctDNA analysis, include the use of paired-end tagging amplification [26] or nanomaterials as electrode modifiers (rGO-CMC [22]), tracing tags (Fe_3_O_4_/TMC/Au [27]), or carriers of signaling molecules (LPA nanoparticles [31]). These strategies offer excellent performance but require quite complex and time-consuming procedures. However, other simpler, faster, and requiring fewer reagents methodologies have been proposed using label [23] or enzymatic-free [25] approaches. It is worth noting also the incorporation of novel technologies, such as EFIRM, based on csw E-field [21], allowing a considerable reduction of both, test times and manipulation steps. 

The selected methods, involving the use of different electrodes substrates (conventional or SPEs), formats (MBs-based or integrated), affinity bioassays (direct and sandwich hybridization, as well as immune-DNA approaches) and electrochemical techniques (DPV, chronoamperometry, EIS, SWV) are just a small sample of the versatility and attractiveness of electrochemical biosensors in the clinic to perform single or simultaneous determination of circulating tumor DNA features by targeted or untargeted analysis. Electrochemical biosensors have been applied mostly to the determinations of tumor-specific mutations and methylation events, and less to ctDNA quantification and cancer-related viral DNA sequences. As shown in Table 1, electrochemical biosensors exhibit wide ranges of sensitivity (LODs ranging from aM [20,24] to μM [32]) and assay times (from less than 10 min [21] to more than 9 h [32]), selectivity even for discrimination of single point mutation [22,23], and methylation processes at single-base resolution [28]. They show also promising practical applicability for the determination in genomic DNA, that has been extracted from a limited number of clinical samples, mostly saliva and plasma, from cancer patients or even directly in serum without previous DNA extraction [30].

It is worth remarking here that electrochemical biosensors offer interesting advantageous features compared with the other most common type of biosensors proposed for ctDNA detection based on optical detection. Although optical reporters can be directly conjugated to the synthetic probe and are less prone to inhibitory effects than the enzyme labels often used in electrochemical strategies, they use bulky, expensive, high-power requirement detectors. On the contrary, electrochemical biosensors overcome the limitations of the optical ones in terms of cost, portability, and compatibility with POC devices, which make them very promising tools, particularly in clinical diagnosis. Moreover, due to the inherent miniaturization of electrochemical biosensors, and their compatibility with standard microfabrication and semiconductor technologies, simple, accurate, portable, and inexpensive platforms can be constructed for ctDNA analysis. It is important also to mention that the strong optical background, that arises from the scattering and absorbance of complex samples, significantly limits the usefulness of optical biosensors when challenged directly in complex environments. These factors explain, as well, why electrochemical biosensors have been increasingly gaining ground compared with their optical counterparts in ctDNA analysis during the past few years [39,40].

Despite the skyrocketing development of electrochemical biosensors and the great promise of ctDNA-based liquid biopsy for cancer diagnosis, prognosis, and personalized treatment [9], hard work is required to circumvent the several biological and technical hurdles, which have, to date, hindered the establishment of ctDNA analysis in routine clinical practice [2]. As far as we are aware from the published literature, no electrochemical biosensing technology has yet entered into clinical practice for ctDNA analysis. 

Some of the most crucial challenges in ctDNA assessment include its isolation and amplification or sequencing analysis, due to its highly fragmented nature and wide variability in the fragment sizes, which affect the accuracy of the results. Another important biological challenge is the low abundance of ctDNA (≥ 1 genome in 5 mL of plasma). Thus, increased volumes of blood samples are needed to perform the determination [2]. Moreover, half-life of ctDNA in circulation is at most of a couple of hours.

It is important to point out the strong dependence of ctDNA concentration with various pre-analytical factors, including sample source, collection, and processing (time interval between collection and centrifugation, choice of anticoagulant and storage time). All these factors should be standardized and considered to develop reliable ctDNA clinical tests [2,5]. 

The low yield of ctDNA extracted [8], and the loss of small DNA fragments (<100 bp), which may possibly host tumor-specific targetable molecular transformations, like mutations, during the isolation processes, are further important issues to which attention should be paid. Despite the various advances and improvements made in ctDNA purification procedures, considerable quantities are still lost at the purification step [2].

Other important challenges are related to the insufficient knowledge of the tumor microenvironment and the immunologic response to ctDNA released in liquid biopsies, and with the adoption of ctDNA analysis in the clinical environment, where not only the laboratory personnel must get familiar with the newly methodologies, but also the medical personnel must increase their knowledge on the potential of this biomarker, and how to implement it in their diagnostic routine. The widespread use of ctDNA analysis in the clinic requires greater standardization and validation of the methodologies, which would provide an efficient framework for their implementation into clinical practice [6]. 

Nowadays, there are scarce studies on the direct comparison among various ctDNA analytical methodologies. Therefore, appropriate methods for reliable ctDNA quantification and efficient recovery for molecular analysis are unsatisfactorily defined. There is a glaring requirement to develop well-established standard operating procedures for ctDNA analysis, bearing in mind all feasible analytical and pre-analytical factors that may affect the test results [2]. Further development in the standardization of these techniques will make ctDNA a valuable substrate in the field of cancer diagnostics [8].

Regarding clinical validation, the development of a ctDNA biomarker cancer detection and monitoring methodology needs vast clinical studies and sample evidence both, for the efficiency of the method and reliability of the marker. The clinical utility of the developed test by performing independent tests, at multiple centers, using large cohorts of patients for each targeted tumor type [2,6]. Moreover, since more information on the relationships among different liquid biopsy markers will contribute to a better understanding of cancer progression and metastasis mechanisms, the complementary use of multi-markers may be beneficial in improving diagnostic, prognostic, and predictive accuracy, and achieving the sensitivity and specificity required for clinical applications. Active and close collaborations between clinicians, devising therapeutic trials and scientists investigating biomarker developments, are required for selecting appropriate biomarkers panels in liquid biopsies. This will also increase tests on the unique opportunities offered by electrochemical biosensors in translational and clinical trial settings, and thereby, help to fully understand the real-world possibilities of this emerging technology, to and reach unmet challenges in clinical management of cancer patients [3,4,9].

Additional progress of novel methodologies is needed to exploit to the fullest ctDNA’s possibilities for early stage cancer patients and those undergoing post-treatment monitoring. Techniques allowing single molecule resolution may prevail in the foreseeable future, given the quality of statistical information they can supply, compared to bulk measurements [5]. Nevertheless, more work is necessary to upgrade and standardize these methods, or to develop new ones with enhanced characteristics. Advancing sample collection and purification techniques is particularly important to guarantee of the various detection possibilities can be accessed. Although, various electrochemical biosensors account for outstanding detection limits, work is needed to develop assays that are also highly multiplexed, more economical, straightforward, and that can be performed outside the research laboratories, or to improve analytical sensitivity and specificity of the methods that already own these characteristics. Lastly, it should be accepted that this discussion has exclusively dealt with technical limitations. As in many fields, the capability of producing reliable data is necessary, but not enough. Other issues, such as the clinical sensitivity or specificity of a mutation (used in the diagnosis of a disease state), selection of target mutations, medical interpretation of the results, clinical value, and legal/ethical matters must also be considered before ctDNA evolves into a prevailing clinical analyte [5].

In conclusion, measuring tumor DNA dynamics in blood, through ctDNA, is a nascent field garnering indisputable attention because of the benefits it brings to cancer management. Moreover, it is poised to advance rapidly both, by overcoming the caveats and technical issues associated with ctDNA as a biomarker, and in the development of novel technologies surpassing the limitations of the conventional ones for its determination. Within this context, electrochemical biosensors constantly pursued over the last five years for this purpose, are envisioned to be indispensable to propel this field forward, by facilitating the ctDNA adoption in a clinical environment.

## Figures and Tables

**Table 1:** Electrochemical biosensing strategies for determination of ctDNAs and specific features.

Electrode	Fundamentals	Target	Technique	Linear range	LOD	Assay Time	Sample	Ref.
***Determination of ctDNA***								
AuE	THMS probe and TdT and RNase HII dual amplification	*KRAS G12DM* ss-ctDNA	DPV (MB)	0.01 fM-1 pM	2.4 aM	~6.5 h + Cp-AuE (4 h)	DNA extracted from plasma of CRCP and HD	[20]
***Tumor-specific mutations in ctDNA***								
Array of 16 bare AuE chips	Sandwich hybridization format using paired capture and FITC-Dp further conjugated with HRP-anti-FITC Fab fragments	*EGFR* mutations	Chronoamperometry (TMB/H_2_O_2_)	—	—	≤ 10 min	Saliva and plasma samples of NSCLC patients	[21]
rGO-CMC-modified SPCE	Direct hybridization using an amino and biotin dually labeled hairpin specific DNA Cp	Single base mutation in *TP53*	Amperometry (TMB/H_2_O_2_)	0.01–0.1 μM	2.9 nM (29 fmol in 10 μL)	45 min + Cp-SPCE (2 h 15 min)	Spiked untreated human serum and saliva samples and cDNA from MCF-10A, MCF-7 and SK-BR-7 cells	[22]
GCE	Direct hybridization at Cp/PEG/AuNPs/GCE	Single base mutation in *BRCA1*	EIS ([Fe(CN)_6_]^3-/4-^)	50.0 fM–1.0 nM	1.72 fM	2 h + Cp/PEG/AuNPs/GCEs (33 h)	Spiked human serum samples	[23]
AuE	HCR at Cp-AuE	Single base mutation in *BRCA1*	DPV (RuHex)	1 aM–10 pM	1 aM	4 h 45 min + Cp-AuE (3 h)	Spiked human serum samples	[24]
NMEs modified with PNA probes	Direct hybridization in connection with a clutch probe strategy	Single base mutation in *KRAS* and *BRAF* in ctDNAs	DPV (Ru(NH_3_)_6_^3+^/Fe(CN)_6_^3−^)	—	0.01% mutation in wild-type DNA	50 min + PNA probes-NMEs (12 h)	ctDNA from serum collected from lung cancer and melanoma patients	[25]
***Epigenetic changes in ctDNA***								
AuE	Paired-end tagging amplification	5-mC (—)	Chronoamperometry (H_2_O_2_/TMB)	—	40 pg (genomic DNA)	~1.5 h (once the modified electrode was prepared)	gDNA extracted from plasma of NSCLC patients	[26]
SPCE	Immunosensor and DNA Dp-modified Fe_3_O_4_/TMC/Au nanocomposite as tracing tags	5-mC/*RASSF1A*	DPV (AuNPs)	1 × 10^−14^-5 × 10^−9^ M	2 × 10^−15^ M	2 h 40 min (once the PT/anti-5mC -SPCE was prepared)	Spiked plasma	[27]
AuE	Bisulfite + self-assembled tetrahedral DNA probes to capture amplicons generated by aMSP	5-mC/*p16INK4a*	Chronoamperometry (H_2_O_2_/TMB)	3–150 pg synthetic target methylated DNA	One methylated DNA molecule in the presence of a 1000-fold excess of unmethylated alleles	aMSP (~42 min) + 45 min (once the AuE was modified with the tetrahedral probes)	cDNA extracted from plasma samples of lung cancer patients	[28]
SPCE	Immunopurification (anti-5-mC-MBs) Immunodetection(b-DNA-Cp-MBs)	5-mC/global (anti-5-mC) and gene-specific (b-DNA-Cp-MBs, *MGMT* and *RASSF1A*)	Amperometry (H_2_O_2_/HQ)	Global (anti-5-mC-MBs): 23−24,000 pM Gene-specific (b-DNA-Cp-MBs): 139−5000 pM (*RASSF1A*) 87–2500 pM (*MGMT*)	Global (anti-5-mC-MBs): 6.8 pM Gene-specific (b-DNA-Cp-MBs): 42 pM (*RASSF1A)* 26 pM (*MGMT*)	Global: 45 min (once the anti-5-mC-MBs were prepared) Gene-specific: 1 h (once b-DNA-Cp-MBs were prepared)	Spiked urine, plasma and saliva	[29]
SPCE	Immunopurification (anti-5-mC or anti-5-hmC-MBs)	5-mC and 5-hmC/global and gene-specific (*MGMT* and *RASSF1A*)	Amperometry (H_2_O_2_/HQ)	Global: 5-mC: 14–2500 pg 5-hmC: 0.04–0.55% Gene-specific: 5-mC: 4.0−250 pM (*MGMT*) 5-hmC: 1.44−100 pM (*MGMT*)	Global: 5-mC: 4.0 pg 5-hmC: 0.004% Gene-specific: 5-mC: 1.2 pM (*MGMT*) 5-hmC: 0.43 pM (*MGMT*)	Global: 45 min (once the anti-5-mC or anti-5-hmC-MBs were prepared) Gene-specific: 90 min (once the anti-5-mC or anti-5-hmC-MBs were prepared)	gDNA extracted from cell lines paraffin-embedded tissues from CRCP and direct determination 1/5 diluted serum from breast and lung cancer patients	[30]
SPCE	Sandwich structure based on PNA probe and anti-5-mC antibody AuNPs and LPA for double signal amplification	Tumor-specific mutations and 5-mC methylation of *PIK3CA* gene	SWV (lead ions)	50 fM–10000 fM	10 fM	1 h 5 min + PNA-AuNPs conjugates (68.5 h) + LPA-anti-5-mC bioconjugates (6 h)	Spiked human plasma samples	[31]
***Cancer-related viral DNA sequences***								
NPGE	Sandwich hybridization approach involving a thiolated Cp and amino-labeled Dp further conjugated with Fc	HBV DNA	DPV (Fc)	3 × 10^−5^–1 × 10^−3^ M	0.8 μM	9 h 20 min + Cp-NPGE (1 h 15 min)	Blood samples from infected people	[32]

aMSP: asymmetric methylation-specific PCR; AuE: gold electrode; AuNPs: gold nanoparticles; cDNA: circulating DNA; Cp: capture probe; CRCP: colorectal cancer patients; Dp: detector probe; DPV: differential pulse voltammetry; EGFR: epidermal growth factor receptor; EIS: electrochemical impedance spectroscopy; Fc: ferrocene; FITC: fluorescein isothiocyanate; GCE: glassy carbon electrode; gDNA: genomic DNA; HBV: Hepatitis B virus; HCR: hybridization chain reaction; HD: healthy donors; 5-hmC: 5-hydroxymethylcytosine; HQ: hydroquinone; HRP: horseradish peroxidase; LOD: limit of detection; LPA: lead phosphate apoferritin; MB: methylene blue; MBs: magnetic beads; 5-mC: 5-methylcytosine; NME: nanostructured microelectrode; NPGE: nanoporous gold electrode; NSCLC: non-small cell lung cancer; PEG: polyethylene glycol; PNA: peptide nucleic acid; PT: polythiophene; rGO-CMC: reduced graphene oxide−carboxymethylcellulose; RuHex: Ru(NH_3_)_6_^3+^; SPCE: screen-printed carbon electrode; SWV: square wave voltammetry; TdT: terminal deoxynucleotidyl transferase; THMS: triplehelix molecular switch; TMB: 3,3´,5,5´-tetramethylbenzidine; TMC: N-trimethyl chitosan.
